# Supplementary material for: Growth anisotropy of the extracellular matrix shapes a developing organ
Source: Nat Commun. 2023 Mar 3;14:1220. doi: 10.1038/s41467-023-36739-y (PMC9984492; doi:10.1038/s41467-023-36739-y)
Supplement: Supplementary file 1 — Supplementary Information [file 41467_2023_36739_MOESM1_ESM.pdf]

Growth anisotropy of the extracellular matrix shapes a developing organ

Harmansa *et.al.*

Supplementary Figures

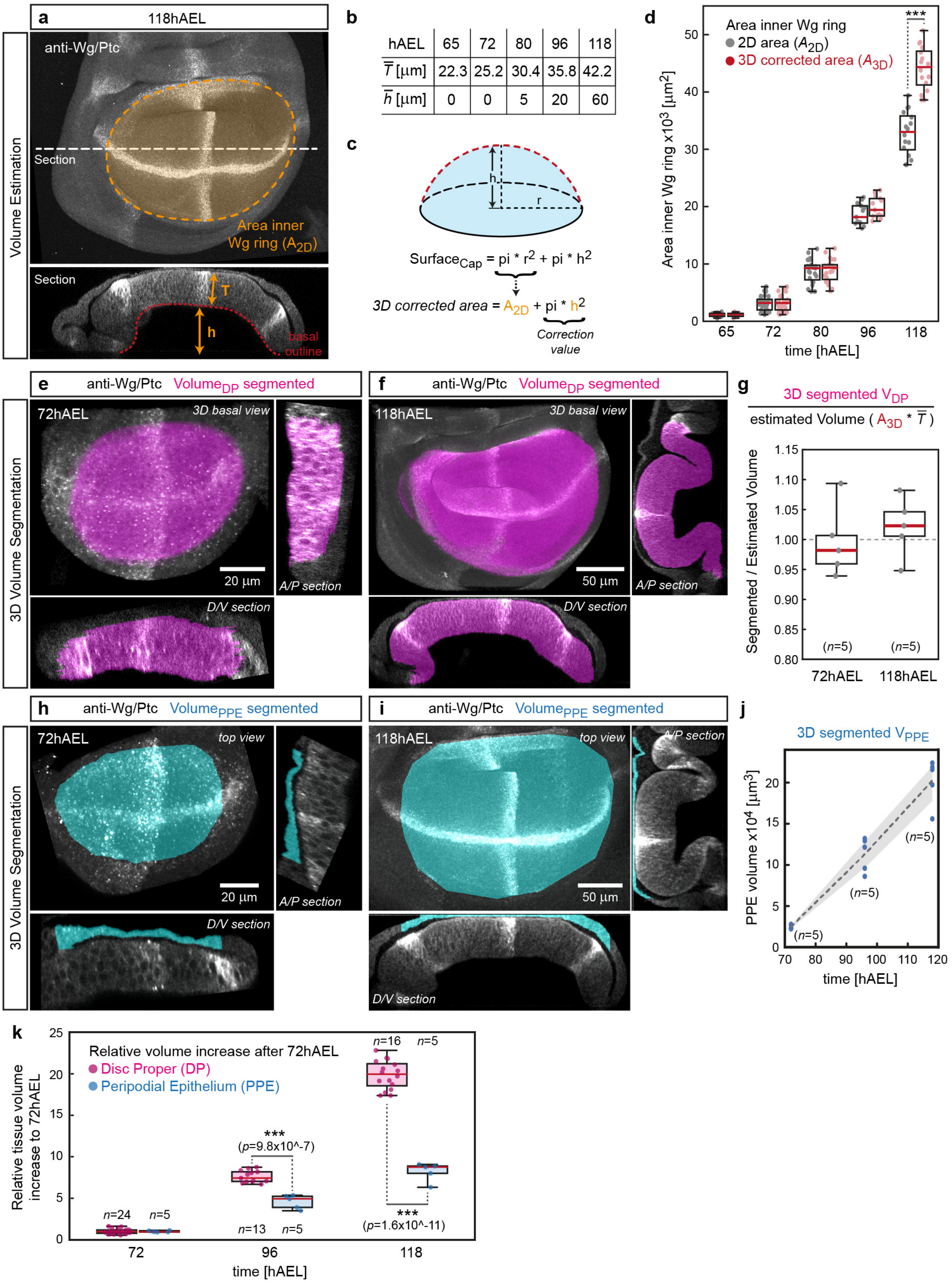

Supplementary Figure 1 – Measuring volume growth of the disc proper and peripodial epithelium

(**a**) The wing pouch area is indicated by the inner Wg ring (A_{2D}, orange dashed line). In cross-section view (bottom) thickness T and height h are indicated. (**b**) Average values for T and h (obtained from profiles in Fig.1b'). (**c**) Using a spherical cap we corrected the 2D area (A_{2D}) for doming after 80hAEL. Spherical cap surface is calculated by the base area (A_{2D}) and height h. Therefore, 2D measured area values are corrected by pi*h^2. (**d**) Wing pouch area measured in 2D (A_{2D}, grey) and corrected for doming (A_{3D}, red). (n_{65}=21 discs, n_{72}=24, n_{80}=23, n_{96}=13, n_{118}=16). (**e+f**) Precise values of wing pouch volume we obtained by volumetric segmentation of the DP tissue encircled by the inner Wg ring (see methods) at 72hAEL (**e**) and 118hAEL (**f**). 3D segmented volume is marked in magenta in plane and section views. (**g**) 3D segmentation requires significant manual correction work. We therefore evaluated if estimation of DP volume (A_{3D}*T) represents the DP volume obtained by 3D segmentation. Indeed, estimated values are not significantly different from volume segmentation values (average error only ~4%). Therefore estimation of DP volume (A_{3D}*T) yields precise values that allow investigating volumetric growth rates of the DP tissue. (**h-i**) Segmentation of peripodial volume overlaying the wing pouch. In example discs at 72hAEL (h) and at 118hAEL (i) the segmented PPE volume is marked (cyan). (**j**) PPE Volume between 72 to 118hAEL (error band represents standard deviation). (**k**) Relative tissue volume increase of the DP (magenta) and the PPE (blue) relative to their volume at 72hAEL. DP volume increases by ~19.8-fold while PPE volume increases by ~8.2-fold. *n*-numbers indicate discs. Statistics: Statistical significance was assessed by a two-sided Student's *t*-test (unequal variance, *$p \leq 0.05$, **$p \leq 0.005$, ***$p \leq 0.0005$). In box plots the median is indicated by a central thick line, while the interquartile range (containing 50% of the data points) is outlined by a box. Whiskers indicate the minimum and maximum data range. Source data are provided as a Source Data file.

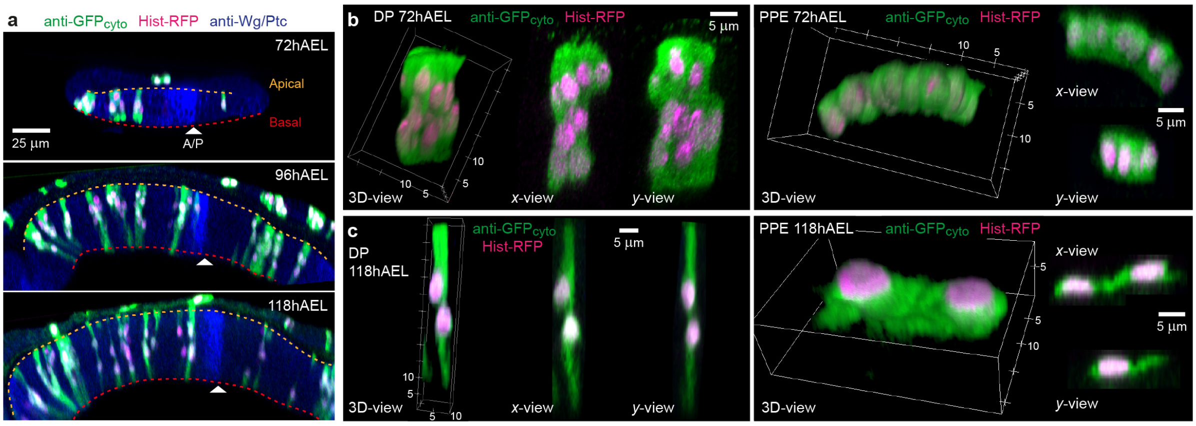

Supplementary Figure 2 – Cell shape changes associated with tissue doming

(**a**) Section view of discs at 72hAEL (*top*), 96hAEL (*middle*) and 118hAEL (*bottom*) expressing cytosolic GFP (*GFPcyto*, green) and *Histone::RFP* (*Hist::RFP*, magenta) in clones of cells. Clones were induced 24h before dissection, e.g. at 48hAEL for the 72hAEL sample. The apical and basal surfaces of the DP are marked by dotted lines (yellow and red, respectively). (**b**) Representative clones in the DP epithelium (*left*) and the PPE (*right*) at 72hAEL (24h after induction). The cell volume is marked by cytosolic GFP (*GFPcyto*, green) and nuclei are labelled by *Hist::RFP* (magenta). Each panel shows a 3D view of the clone (left) and section views in *x*- and *y*-direction (middle-right). (**c**) Same as in (b) but at the end of larval development at 118hAEL.

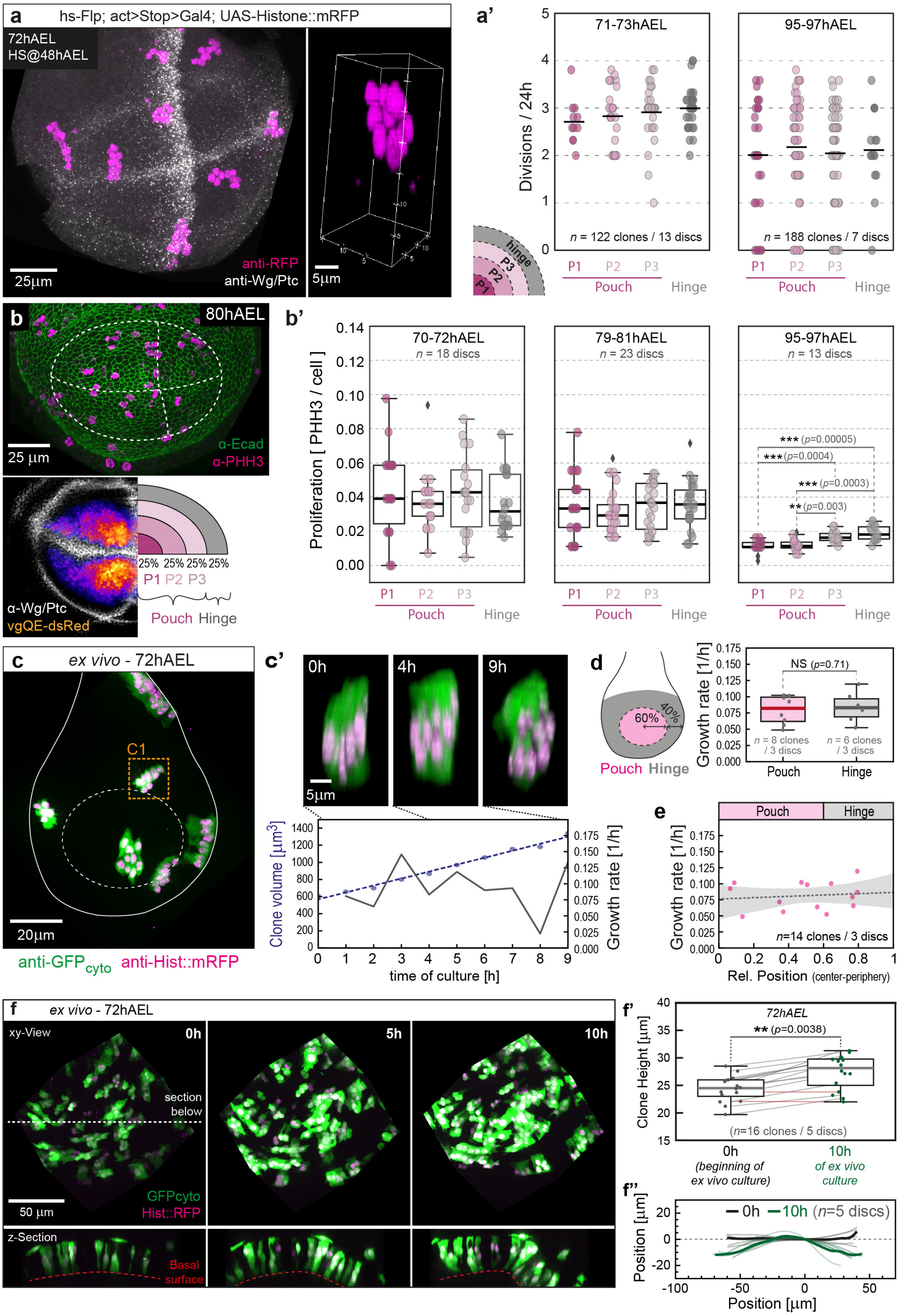
a
hs-Flp; act>Stop>Gal4; UAS-Histone::mRFP
72hAEL
HS@48hAEL
anti-RFP
anti-Wg/Ptc
25µm
5µm
a'
71-73hAEL
95-97hAEL
Divisions / 24h
n = 122 clones / 13 discs
n = 188 clones / 7 discs
P1 P2 P3
Pouch
Hinge
b
80hAEL
25 µm
α-Ecad
α-PHH3
α-Wg/Ptc
vgQE-dsRed
25% 25% 25% 25%
b'
70-72hAEL
n = 18 discs
79-81hAEL
n = 23 discs
95-97hAEL
n = 13 discs
Proliferation [PHH3 / cell]
*** (p=0.00005)
*** (p=0.0004)
*** (p=0.0003)
** (p=0.003)
c
ex vivo - 72hAEL
C1
20µm
anti-GFPcyto anti-Hist::mRFP
c'
0h
4h
9h
Clone volume [µm3]
Growth rate [1/h]
time of culture [h]
d
60%
40%
NS (p=0.71)
n = 8 clones / 3 discs
n = 6 clones / 3 discs
e
Rel. Position (center-periphery)
n=14 clones / 3 discs
f
ex vivo - 72hAEL
xy-View
0h
5h
10h
section below
50 µm
GFPcyto
Hist::RFP
z-Section
Basal surface
f'
72hAEL
** (p=0.0038)
Clone Height [µm]
(n=16 clones / 5 discs)
0h (beginning of ex vivo culture)
10h of ex vivo culture
f''
0h 10h (n=5 discs)
Position [µm]

Supplementary Figure 3 - Growth is homogeneous in the plane of the DP epithelium
(**a**) 72hAEL wing disc expressing Histone::RFP in clones (24h after heat shock (HS)). *right*: 3D-volume view of example clone. (**a'**). Clonal division rates at 72hAEL (*left*) and at 96hAEL (*right*) assessed in 4 elliptic domains covering the pouch (P1-P3, central to peripheral) and the hinge (as shown *bottom left*). (**b**) *top*: 80hAEL old wing disc stained for E-Cadherin (green) and Phospho-Histone H3 (magenta). Wg/Ptc cross is indicated by dashed lines. *bottom*: 80hAEL wing disc expressing dsRed under control of the *vestigial Quadrant enhancer* (*vgQE-dsRed*, a marker for the wing pouch) co-labelled for Wg/Ptc. The inner Wg ring was subdivided into 4 domains covering the wing pouch tissue (P1-P3, shades of purple) and the peripheral hinge tissue (grey). (b') Proliferation density based on PHH3 staining (see panel b) and cell density. (**c**) *ex vivo* cultured 72hAEL wing disc expressing clones of cytosolic GFP (clone volume) and Histone::RFP (nuclei) at beginning of culture. (c') A magnified clone (C1) is shown in 3D-view at indicated times of culture. *bottom*: Clonal volume and growth rate over 9h of culture. (**d**) *left:* Clones were classified as 'pouch' or 'hinge', depending on their position. *right*: Average clonal growth rate at 72hAEL. (**e**) Data shown in (d) plotted in respect to relative clonal position (centre to periphery). A linear regression is shown by a grey dashed line (error band indicates standard deviation). (**f**) Representative wing disc containing clones marked by cytosolic GFP (GFP_{cyto}) and Histone::RFP (Hist::RFP), dissected at 72hAEL and cultured *ex vivo* for indicated hours in plane (*top*) and section view (*bottom*). The basal outline is marked by a red dashed line in the section view. Explants of 72hAEL old larvae are relatively flat at beginning of culture (left) and bend during the culture period. (**f'**) Quantification of clone height for 72hAEL at beginning of culture (0h) and after 10h of *ex vivo* culture (10h). Cell height significantly increases during the 10h culture period. Only two clones did not increase in height (red lines). (**f''**) Average basal outline of five 72hAEL discs at beginning (black) and after 10h (green) of *ex vivo* culture. Statistics: Statistical significance was assessed by a two-sided Student's *t*-test (unequal variance, *$p \leq 0.05$, **$p \leq 0.005$, ***$p \leq 0.0005$). In box plots the median is indicated by a central thick line, while the interquartile range (containing 50% of the data points) is outlined by a box. Whiskers indicate the minimum and maximum data range. Source data are provided as a Source Data file.

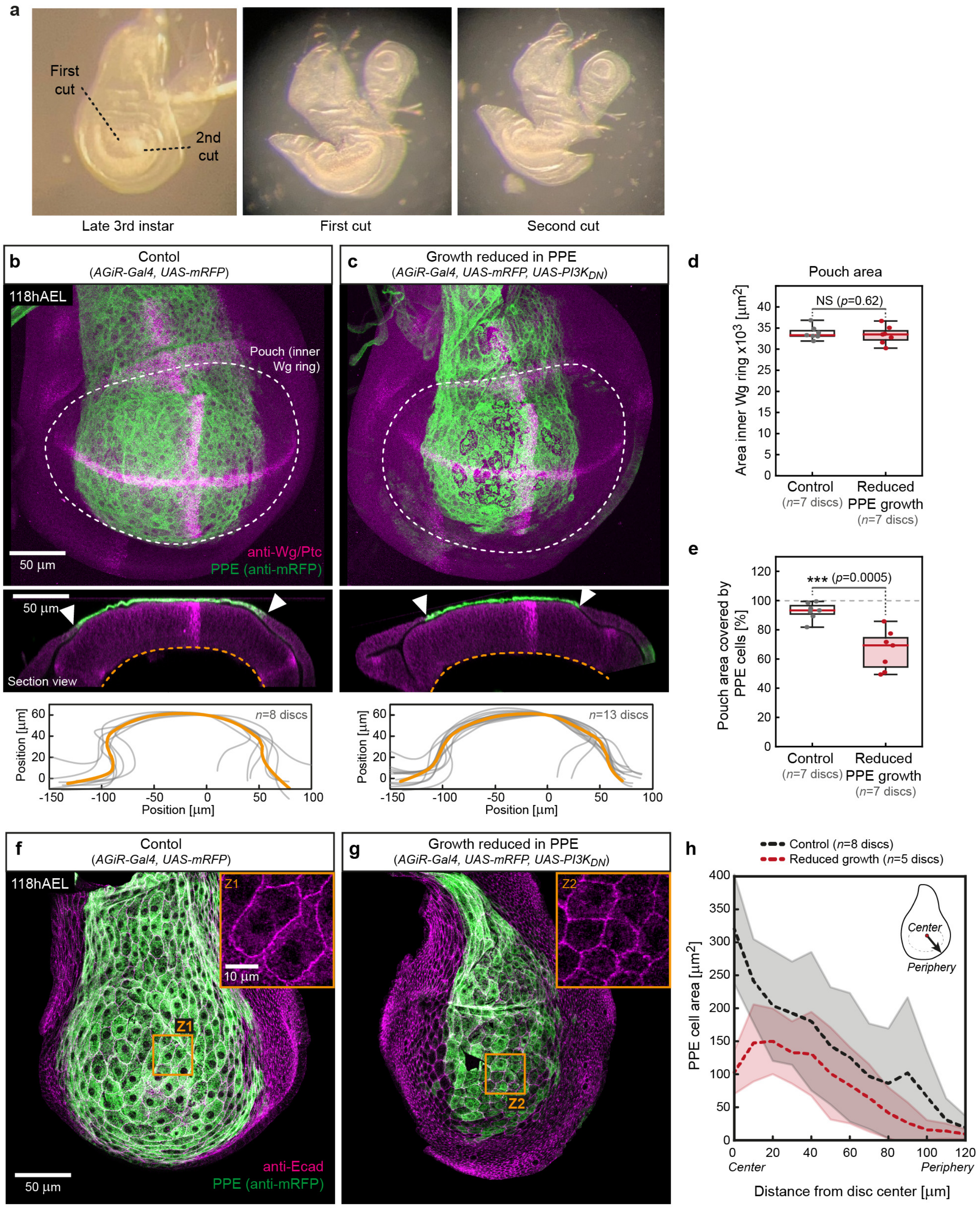

Supplementary Figure 4 – Growth modifications in the peripodial epithelium

(**a**) Wing disc explant before (*left*) and after 2 sequential cuts performed with a micro-scissor (*middle, right*). The cuts are indicated by dashed lines (*left*). (**b**) Peripodial projection (*top*) and section view (*middle*) of an 118hAEL wing disc expressing RFP in peripodial cells (*AGiR-Gal4*). In controls, the RFP-marked PPE covers large parts of the wing pouch (dashed line). The extent of the PPE is marked by two arrowheads in the section view below. Quantifications show that these discs form a dome of ~60µm height (bottom) as observed previously (see Fig.1b'). (**c**) Overexpression of a dominant-negative form of PI3K ($PI3K_{DN}$) in peripodial cells results in reduced cellular growth and reduced PPE area (smaller green domain). Despite reduced PPE growth discs still form a dome of similar extent as control discs (*bottom*). (**d**)

Quantifications of pouch area in control discs (b) and in discs with reduced PPE growth (c) show that peripodial expression of $PI3K_{DN}$ does not affect DP growth. (**e**) Quantification of the pouch area (inner Wg ring) covered by mRFP positive peripodial cells. While in control discs the peripodial epithelium covers ~95% of the pouch, peripodial $PI3K_{DP}$ overexpression significantly reduces pouch coverage to ~66%. (**f-g**) Peripodial projection of control (e) and $PI3K_{DN}$ expressing discs (f) stained for E-cadherin (Ecad) to visualize cell outlines. Magnifications of the central PPE region marked by orange rectangles are shown top right (Z1 and Z2). (**h**) Quantification of peripodial apical cell area in control disc (black) and in disc where PPE growth was reduced by $PI3K_{DN}$ (red) as shown in (f-g). In control discs peripodial cell area is maximal in the centre of the disc and decreases towards the periphery. In $PI3K_{DN}$ discs the central PPE cell area is significantly reduced. (Error bands indicate standard deviation). Statistics: Statistical significance was assessed by a two-sided Student's *t*-test (unequal variance, *$p \leq 0.05$, **$p \leq 0.005$, ***$p \leq 0.0005$). In box plots the median is indicated by a central thick line, while the interquartile range (containing 50% of the data points) is outlined by a box. Whiskers indicate the minimum and maximum data range. Source data are provided as a Source Data file.

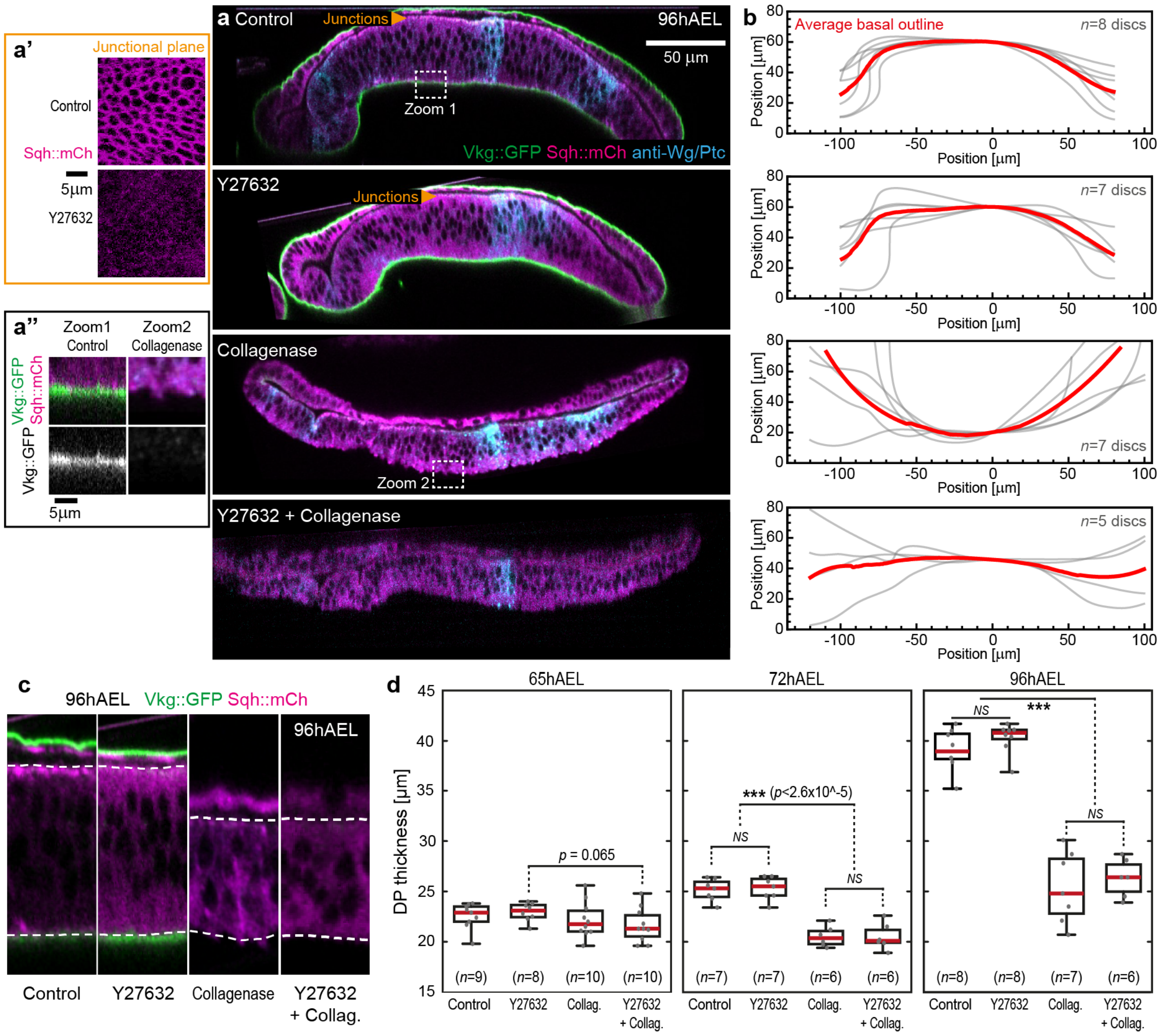

Supplementary Figure 5 – Acute digestion of the ECM concomitant with MyoII inhibition
(**a**) Optical cross-sections parallel to the D/V boundary of representative *Vkg::GFP* (ECM) and *Sqh::mCherry* (MyoII) wing discs at 96hAEL upon the following treatments: *top:* not-treated control, *middle-top*: Rock inhibitor Y27632 treated (MyoII inhibition), *middle-bottom*: Collagenase treated (loss of the ECM) and *bottom*: double treated (Y27632+Collagenase). *Sqh::mCherry* signal in the junctional plane (see orange arrow heads) is observed in-plane-views in control discs (a' *top*) but lost upon treatment with Y27632 (a' *bottom*). The ECM labelled by Vkg::GFP (see a'' zoom1 for control ECM) is completely lost upon treatment with Collagenase (a'' zoom2). (**b**) Quantifications of the average basal outline of the DP epithelium for the conditions shown in (a). Individual profiles are shown in grey, the average profile in red. While control and Y27632 treated discs maintain domed morphology, the loss of the ECM layer (by Collagenase treatment) results in significant deformation of the epithelial layers: In the presence of MyoII activity (*middle-bottom*) discs tend to inverse their shape and bend upwards. In contrast, a loss of both, MyoII and the ECM results in a nearly flat and relaxed epithelial layer. (**c**) Representative cross sections of the region close to the A/P-D/V intersection of the indicated treatments. The apical and basal surface of the DP epithelium is indicated by dashed lines. (**d**) Quantification of DP epithelial thickness close to the A/P-D/V intersection for different developmental time-points and treatments. While the inhibition of MyoII does not significantly affect DP thickness at any time-point, a loss of the ECM results in a significant reduction in DP thickness at 72 and 96hAEL (in both the Collagenase and the

Y27632+Collagenase treated discs). *n* indicates number of discs. Statistics: Statistical significance was assessed by a two-sided Student's *t*-test (unequal variance, *$p \leq 0.05$, **$p \leq 0.005$, ***$p \leq 0.0005$). In box plots the median is indicated by a central thick line, while the interquartile range (containing 50% of the data points) is outlined by a box. Whiskers indicate the minimum and maximum data range. Source data are provided as a Source Data file.

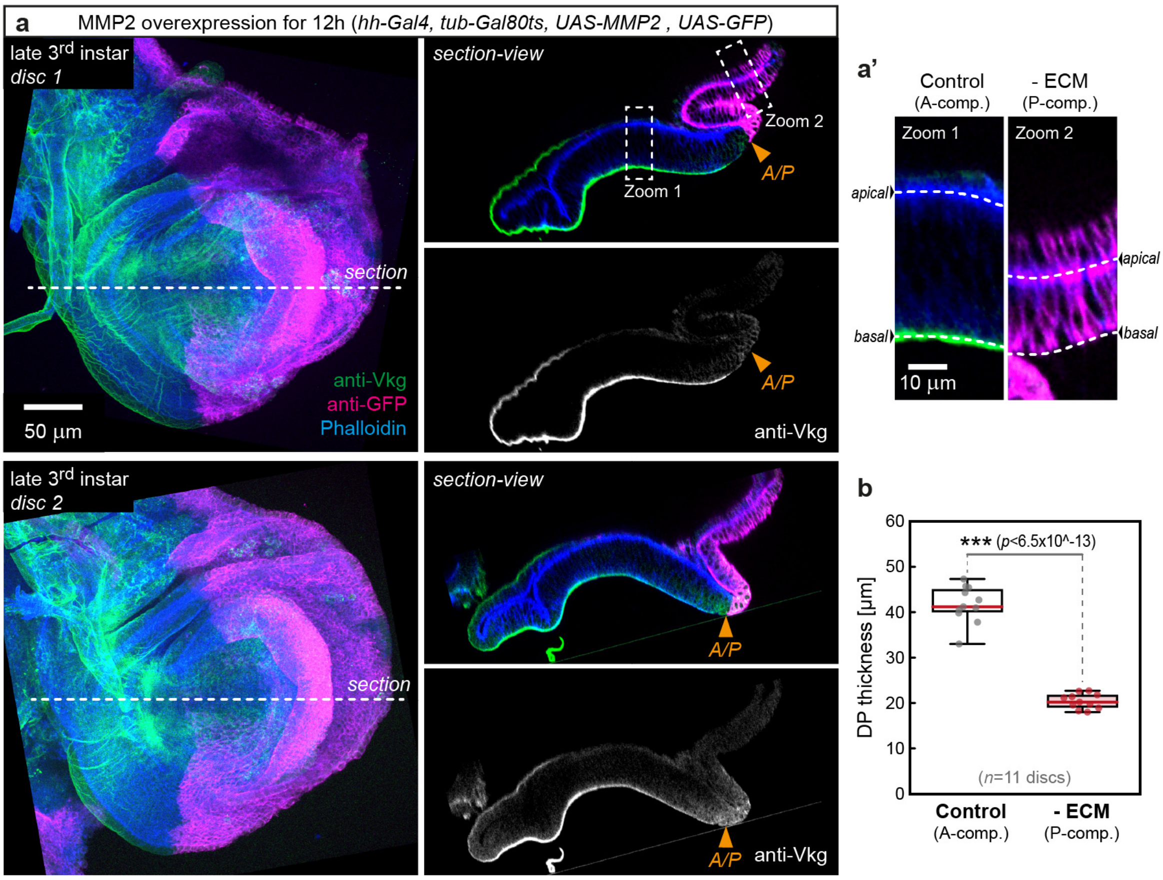

Supplementary Figure 6 – Genetic degradation of the ECM results in epithelial thickness relaxation

(**a**) Two representative late 3rd instar wing discs (*disc 1* and *disc 2*) overexpressing Matrix-Metalloproteinase 2 (MMP2) in the posterior compartment (P-comp., magenta) for 12 hours before dissection in plane (left) and cross-section view (right). Temporal induction of MMP2 expression was controlled by a temperature shift to 29C thereby inhibiting a temperature sensitive version of the Gal80 (*Gal80ts*) protein. Discs were stained for Vkg (green) to visualize the ECM and F-Actin (Phalloidin) to mark epithelial outlines. While the posterior overexpression of MMP2 results in a loss of the ECM in the P-comp. and epithelial thickness relaxation, the anterior compartment (A-comp.) maintains its ECM and normal epithelial shape serving as internal control. (**a'**) Magnification of the DP epithelium in the anterior versus the posterior compartment as indicated in (a, *right*: zoom 1 and zoom 2). (**b**) Quantification of epithelial thickness in the A-comp. (control) versus the P-comp. (no ECM). Genetic digestion of the ECM by MMP2 overexpression results in a reduction of epithelial thickness to ~20µm, a value very close to the reference thickness (~21.5µm) we observed upon acute digestion of the ECM using Collagenase (see Fig.3 and Supplementary Figure 5d). Therefore, the DP epithelium does not grow actively in z-direction but increase in tissue thickness are due to ECM mediated cell crowding and compression. Statistics: Statistical significance was assessed by a two-sided Student's *t*-test (unequal variance, $*p \leq 0.05$, $**p \leq 0.005$, $***p \leq 0.0005$). In box plots the median is indicated by a central thick line, while the interquartile range (containing 50% of the data points) is outlined by a box. Whiskers indicate the minimum and maximum data range. Source data are provided as a Source Data file.

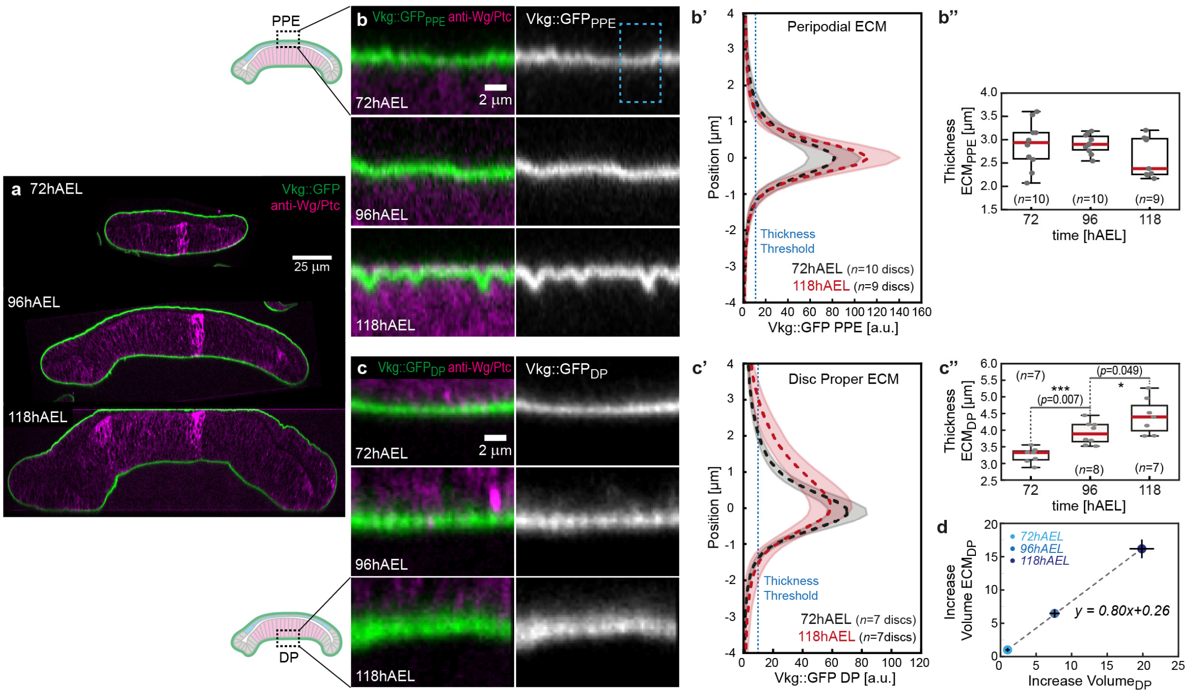

Supplementary Figure 7 – The disc proper epithelium outgrows its ECM layer

(**a**) Cross-sections parallel to the D/V boundary of discs expressing a GFP-tagged version of *Drosophila* Collagen IV (Viking, Vkg::GFP). A shell of ECM (green) can be observed covering the basal side of the epithelial cells at all observed stages. (**b**) Magnifications of representative sections of the peripodial ECM layer (Vkg::GFP$_{PPE}$) at defined stages. Towards the end of development, the ECM$_{PPE}$ tends to be wrinkled. (**b'**) Average plots of Vkg::GFP intensity along the apical-basal axis (as indicated in dashed box in (b)). An increase in Vkg::GFP$_{PPE}$ peak density is observed from 72 to 118hAEL. Error band indicates the standard deviation. (**b''**) However, when ECM$_{PPE}$ thickness is quantified based on an intensity threshold (see dashed blue line in (b'), no change in thickness is observed. See methods for details on intensity profiles and thickness quantifications. (**c**) Representative section of the bottom ECM$_{DP}$ underlining the basal surface of the DP epithelium. A clear increase in thickness is visible from 72 to 118hAEL. This tendency to increase in thickness can be quantified in Vkg::GFP$_{DP}$ apical-basal intensity profiles (c'). A significant increase in thickness is quantified in (c''). Error band indicates the standard deviation. (**d**) Plot of relative DP volume increase versus relative estimated ECM volume increase (obtained by multiplying the area of the inner Wg ring with the ECM thickness obtained in (c'')). We observe a linear correlation with a slope of ~0.8 (dashed line). This result suggests that the DP epithelium outgrows its EMC by ~20%. Error bars indicate standard deviation. Statistics: Statistical significance was assessed by a two-sided Student's *t*-test (unequal variance, **p*≤0.05, ***p*≤0.005, ****p*≤0.0005). In box plots the median is indicated by a central thick line, while the interquartile range (containing 50% of the data points) is outlined by a box. Whiskers indicate the minimum and maximum data range. Source data are provided as a Source Data file. The schemes in panels b and c are adapted from Harmansa *et.al.*, A nanobody-based toolset to investigate the role of protein localization and dispersal in *Drosophila. eLife 6,e22549 (2017)*, licensed under CC BY 4.0.

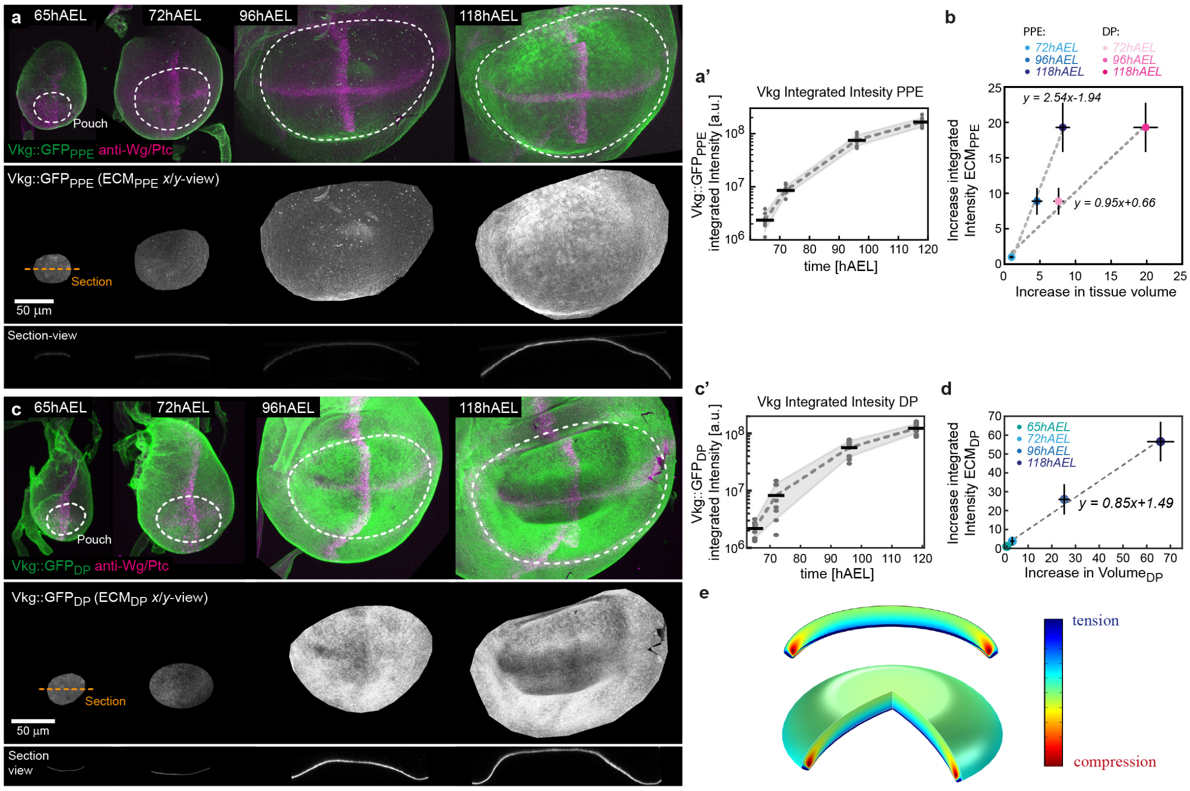

Supplementary Figure 8 – Quantifications of the magnitude of ECM growth

(**a**) *top*: Maximum projections of the peripodial side of discs expressing Vkg::GFP (green), stained for Wg/Ptc (magenta). We have masked and extracted the Vkg::GFP$_{PPE}$ signal (top ECM) covering the DP (inner Wg ring, dashed line). Projections in-plane (*middle*) and cross-section view (*bottom*). (**a'**) The integrated fluorescence intensity of the Vkg::GFP$_{PPE}$ covering the DP (as shown in (a, *middle*)) is proportional to Vkg::GFP$_{PPE}$ levels and hence approximates ECM deposition. Black bar indicates the mean, error band indicates the standard deviation. (Sample numbers: n_{65}=8, n_{72}=11, n_{96}=10, n_{118}=11). (**b**) Relative increase in integrated Vkg::GFP$_{PPE}$ intensity versus volume increase of the peripodial epithelium (blue) and the DP epithelium (magenta, as quantified in Supplementary Fig.1k). Linear regressions are indicated by dashed lines, error crosses indicate standard deviation. Vkg::GFP$_{PPE}$ levels increase faster than the peripodial volume (slope of linear regression ~2.54) suggesting that the top ECM outgrows the peripodial cell layer. In contrast, the integrated Vkg::GFP$_{PPE}$ intensity and the DP volume increase to a similar extent (slope of linear regression ~0.95) from 72 to 118hAEL. (disc numbers PPE: n_{72}=11, n_{96}=10, n_{118}=11, disc numbers DP: n_{72}=9, n_{96}=11, n_{118}=12). (**c**) *top*: Maximum projections of the DP side of Vkg::GFP discs (green), stained for Wg/Ptc (magenta) at indicated stages. *middle/bottom*: Masked and extracted Vkg::GFP$_{DP}$ signal within the inner Wg ring underlining the DP. Projections in-plane (*middle*) and cross-section view (*bottom*). (**c'**) Integrated fluorescent intensity of the Vkg::GFP$_{DP}$ underlining the DP (as shown in (c)). (**d**) Relative volume increase of the disc proper epithelium plotted versus the relative increase in integrated Vkg::GFP$_{DP}$ intensity (linear correlation, slope of ~0.85). This implies that the DP outgrows the ECM$_{DP}$ layer (also see Supplementary Fig.7d). (Linear regression is marked by dashed line, n_{72}=9, n_{96}=11, n_{118}=12, error cross indicates standard deviation) (**e**) Assuming that the ratio of pouch volume increase divided by the ECM$_{DP}$ volume increase between 65h and 118h is 0.79, and that the growth of both layers is planar, we find that in our simulations this growth mismatch is not sufficient to describe the correct tissue geometry. Source data are provided as a Source Data file.

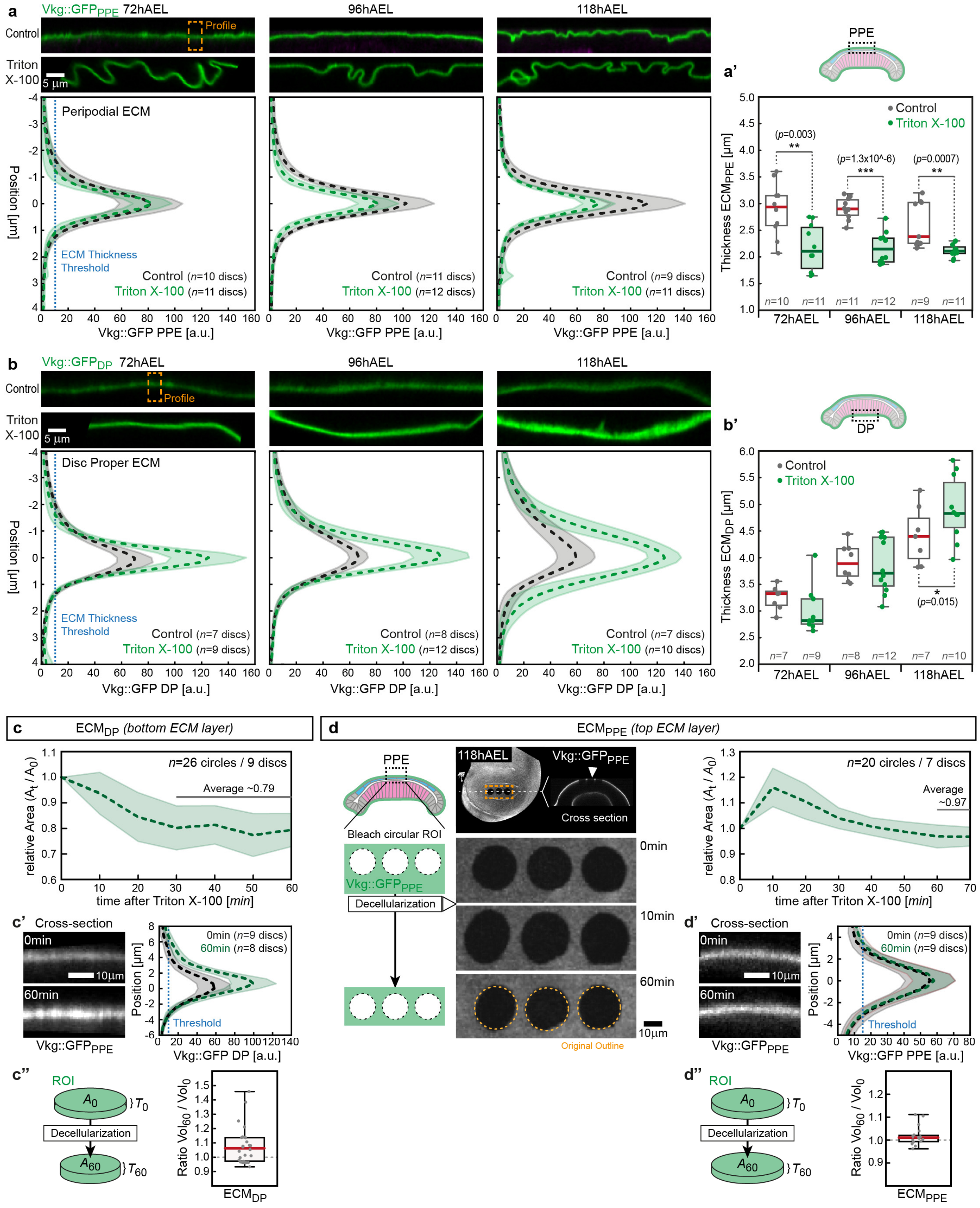

Supplementary Figure 9 – Changes in ECM morphology during larval growth (decellularization)

(**a**+**b**) ECM changes upon decellularization in fixed samples at different developmental stages in the PPE (a) and the DP (b). Fluorescent intensities and profiles are comparable between different timepoints and conditions. (**a**) *top-middle*: Cross-sections of the peripodial ECM labelled by Vkg::GFP in control (*top*) and decellularized (*middle*, Triton X-100 treated) wing discs at indicated age classes. *Bottom*: Profiles of peripodial Vkg::GFP levels (ECM_{PPE}) in control (black) and decellularized conditions (green) Error bands indicate standard deviation. (**a'**) Quantification of peripodial ECM thickness at indicated time-points in control (black) and

decellularized discs (green). During development we do not observe a significant change in ECM thickness, however, upon decellularization ECM thickness decreases. *n* indicates number of discs. (**b**) Same as in (a) but for the bottom ECM_{DP}. (**b'**) In contrast to the peripodial ECM, the thickness for the bottom ECM_{DP} increases significantly during development. (**c**) Bottom ECM_{DP} relative circular area plotted over time after the addition of Triton X-100 in *ex vivo* culture. The relative area decreases for 30min before reaching a plateau value at ~79% of the original area. (**c'**) *left*: Cross-section of representative sections of the bottom ECM_{DP} before and 60min after Triton X-100 addition. *right*: Quantification of Vkg::GFP_{DP} intensity before and after decellularization. The profile of the relaxed bottom ECM_{DP} shows increased peak intensity and increased width at the threshold value (dotted blue line, 10a.u.) chosen to quantify ECM thickness in Fig.4d''. (**c''**) Quantification of changes in estimated circular ECM volume (area *A* * thickness *T*) before (Vol_0) and 60min after decellularization (Vol_{60}, *n*=26 circles / 9 discs) (**d**) Results for *ex vivo* decellularization in the ECM_{PPE} layer. *left*: Three circular regions of interest (ROIs) were marked by photobleaching onto the ECM_{PPE} of 118hAEL Vkg::GFP wing discs. *right*: Relative area changes after addition of Triton X-100. In contrast to the bottom ECM_{DP}, the relative area in the top ECM_{PPE} transiently increases before reaching a plateau at ~97% of original area after 60-70min. (**d'**) ECM_{PPE} thickness and Vkg::GFP density does not significantly change upon decellularization. (**d''**) Estimated ECM_{PPE} volume marked by Vkg::GFP remains constant upon decellularization (*n*=20 circles / 7 discs). Statistics: Statistical significance was assessed by a two-sided Student's *t*-test (unequal variance, *$p \leq 0.05$, **$p \leq 0.005$, ***$p \leq 0.0005$). In box plots the median is indicated by a central thick line, while the interquartile range (containing 50% of the data points) is outlined by a box. Whiskers indicate the minimum and maximum data range. In line plots the error bands indicate the standard deviation. Source data are provided as a Source Data file. The schemes in panels a',b' and d are adapted from Harmansa *et.al.,* A nanobody-based toolset to investigate the role of protein localization and dispersal in *Drosophila. eLife 6,e22549 (2017)*, licensed under CC BY 4.0.

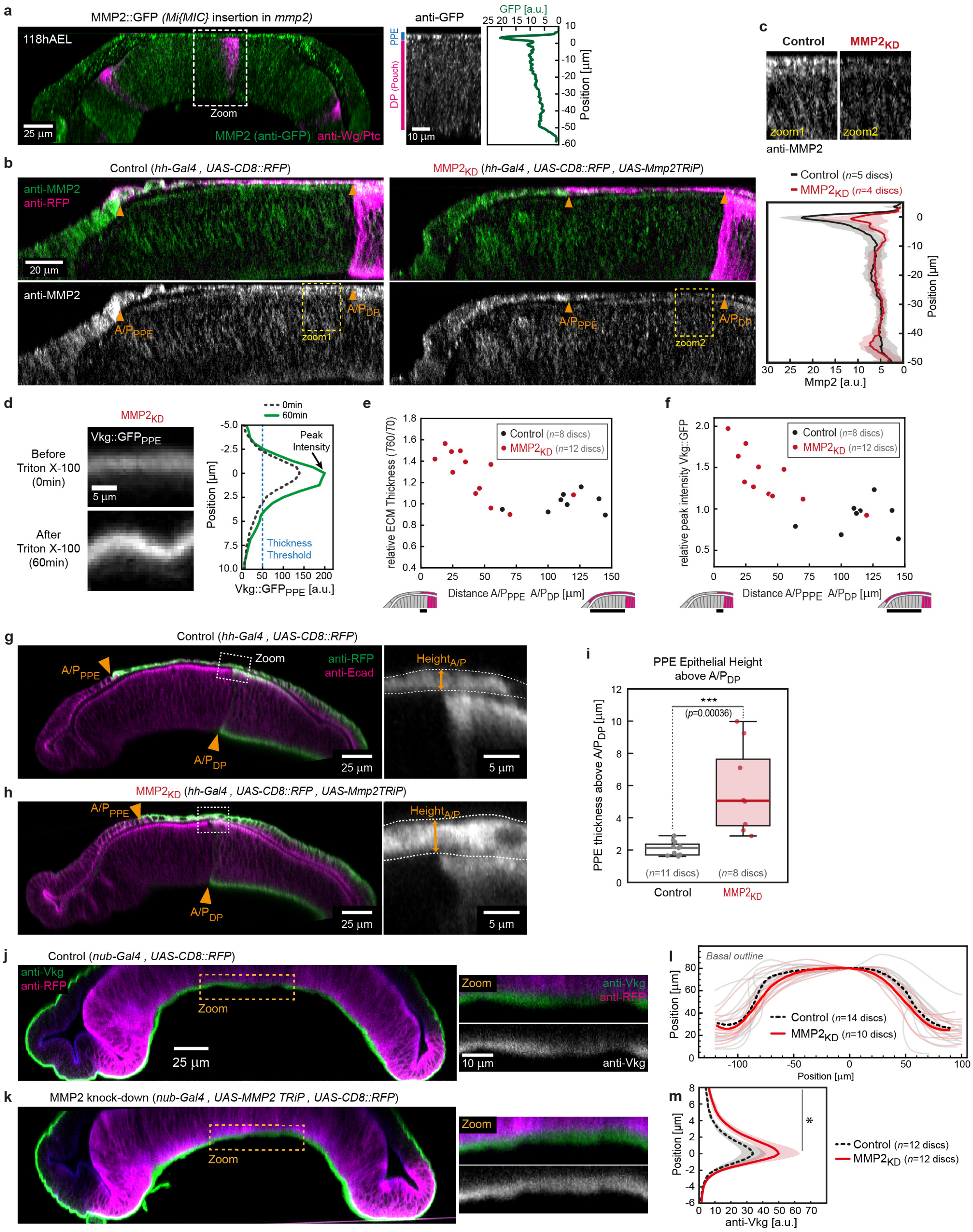

Supplementary Figure 10 - MMP2 localization and TRiP-mediated MMP2 knock-down
(**a**) Cross-section of a 118hAEL wing disc expressing GFP-tagged MMP2 (via Mi{MIC} insertion). MMP2::GFP is predominantly observed in the PPE. *right*: Magnification of indicated region (zoom) and intensity profile of MMP2::GFP. (**b**) *left*: Control wing disc expressing RFP in the posterior compartment (*hh-Gal4*) stained for MMP2 (green). Arrowheads indicate the anterior-posterior (A/P) compartment boundary in the PPE and DP layer. *right*: Knock-down of MMP2 in the posterior compartment results in reduced posterior MMP2 levels. (**c**) *top*: Magnifications of the regions marked in (b). *bottom*: Average MMP2 profiles of control (black) and $MMP2_{KD}$ discs (red). The domain between x=0 to x=-5 corresponds to the PPE (error

bands indicate standard deviation). (**d**) Peripodial ECM posterior of the A/P boundary before (*top*) and after decellularization (*bottom*). Vkg::GFP_{PPE} intensity profiles indicate ECM thickness (threshold value 50 a.u.) and peak intensity (maximum intensity of profile). (**e**) Relative change of ECM_{PPE} thickness ($Thickness_{60min}$ / $Thickness_{0min}$) upon decellularization versus the distance between the peripodial and disc proper A/P boundary. While in control wing discs this distance is typically ~120µm at 118hAEL, in $MMP2_{KD}$ this distance decreases with increasing knock-down efficiency. Consistently, the discs showing the strongest ECM thickness changes also show strong reduction in A/P compartment boundary distance. (**f**) Relative Vkg::GFP peak intensity changes ($Intensity_{60min}$ / $Intensity_{0min}$) upon decellularization plotted against A/P boundary distance. (**g**) Section view of control wing discs expressing RFP (green) in the posterior compartment. *right*, Magnification, peripodial height above the A/P_{DP} is indicated by an orange arrow. (**h**) $MMP2_{KD}$ wing disc expressing RFP and Mmp2 TRiP in the P-compartment. (**i**) Height of peripodial epithelium above the A/P_{DP}. Statistical significance was assessed by a two-sided Student's *t*-test (unequal variance). In box plots the median is indicated by a central thick line, while the interquartile range (containing 50% of the data points) is outlined by a box. Whiskers indicate the minimum and maximum data range. (**j-k**) Late 3rd instar wing disc expressing either CD8::RFP (magenta) alone (j, control) or together with a MMP2 TRiP line (k, $MMP2_{KD}$) in the DP wing pouch (*nub-Gal4*), stained for Vkg (green). Central ECM_{DP} is magnified to the right. (**l**) Quantification of basal disc outline. $MMP2_{KD}$ in the DP results in slightly increased curvature. (**m**) DP knock-down of MMP2 leads to a significant increase in Vkg peak intensity and increased ECM thickness. These observations are consistent with the notion that MMP2 is also required in the DP tissue to fine-tune ECM growth anisotropy and disc shape. The error bands indicate the standard deviation. Source data are provided as a Source Data file. The schemes in panels e and f are adapted from Harmansa et.al., A nanobody-based toolset to investigate the role of protein localization and dispersal in *Drosophila. eLife 6,e22549 (2017)*, licensed under CC BY 4.0.

Supplementary information

Stefan Harmansa* [1] Alexander Erlich* [1,2,3] Christophe Eloy [2]

Giuseppe Zurlo [4] Thomas Lecuit† [1,5]

[1] *Aix-Marseille Université & CNRS, IBDM - UMR 7288 & Turing Center for Living Systems (CENTURI), Campus de Luminy case 907, 13288 Marseille France.*

[2] *Aix-Marseille Université, CNRS, Centrale Marseille, IRPHE, Turing Centre for Living Systems, Marseille, France.*

[3] *Present address: Université Grenoble Alpes, CNRS, LIPHY, 38000 Grenoble, France*

[4] *School of Mathematical and Statistical Sciences, University of Galway, University Road, Galway, Ireland.*

[5] *Collège de France, 11 Place Marcelin Berthelot, Paris, France.*

M1 Description of the multi-layer wing disc model

M1.1 Theoretical framework

To gain insight into the shape of the growing wing disc and estimate the growth-induced stress, we modelled the *Drosophila* wing disc as a growing elastic tissue, implementing an existing theory of tissue growth that has been applied successfully to arteries and brain tissue [9, 13, 12]. The numerical implementation was solved in the finite element methods framework `COMSOL Multiphysics`.

The tissue grows according to a specified growth deformation tensor, $\mathbb{G}$. The total deformation gradient $\mathbb{F}$ consists of two contributions: The growth tensor $\mathbb{G}$ and the elastic deformation gradient $\mathbb{A}$, i.e. $\mathbb{F} = \mathbb{A}\mathbb{G}$. The multiplicative decomposition is explained in more detail in the caption of Fig. M2. We also made the assumption that the tissue is a hyperelastic nearly-incompressible neo-Hookean material [12, 7, 8] with the elastic energy W, given by [1, 9, 6]

$$W = \frac{1}{2}\left[\mu\left(\tilde{I}_1 - 3\right) + \kappa\left(|\mathbb{A}| - 1\right)^2\right] \tag{M1}$$

where μ and κ are the shear and bulk modulus of the material, respectively, and $|\mathbb{A}|$ is the determinant of the elastic deformation gradient $\mathbb{A}$. Further, $\tilde{I}_1 = I_1 |\mathbb{A}|^{-2/3}$, where I_1 is the first invariant of the right Cauchy-Green deformation tensor, $I_1 = \mathrm{tr}(\mathbb{A}^\mathsf{T}\mathbb{A})$. Finally, the Cauchy stress tensor is given by

$$\mathbb{T} = \frac{1}{|\mathbb{A}|}\frac{\partial W(\mathbb{A})}{\partial \mathbb{A}}\mathbb{A}^\mathsf{T}. \tag{M2}$$

To obtain the deformation of the body with a prescribed growth tensor $\mathbb{G}$, we solve the balance of linear momentum div $\mathbb{T} = \mathbf{0}$. The linear momentum balance is coupled with the morphoelastic decomposition $\mathbb{F} = \mathbb{A}\mathbb{G}$, in conjunction with the constitutive law (M2). We assume that the external boundary of the wing disc is traction-free, $\mathbb{T}\mathbf{n} = \mathbf{0}$, where $\mathbf{n}$ is the outward facing surface normal. The wing disc is assumed to be axisymmetric and we enforce a no-displacement boundary condition on one point along the symmetry axis. The full set of boundary conditions is shown in Fig. M1.

In a cylindrical basis $\{\mathbf{E}_R, \mathbf{E}_\theta, \mathbf{E}_Z\}$, we assume that the growth tensor takes the form

$$\mathbb{G} = \gamma_i\left(\mathbf{E}_R \otimes \mathbf{E}_R + \mathbf{E}_\theta \otimes \mathbf{E}_\theta\right) + \gamma_{Z,i}\mathbf{E}_Z \otimes \mathbf{E}_Z, \qquad i \in \{\mathrm{PPE}, \mathrm{DP}, \mathrm{ECM}\}, \tag{M3}$$

where $\gamma_{Z,i}$ represents growth in the direction of the long cylinder axis and γ_i growth in the direction perpendicular to it, i.e. in the circular plane which is the cross-section of the cylinder. If $\gamma_i = \gamma_{Z,i}$,

*These authors contributed equally: Stefan Harmansa, Alexander Erlich

†Correspondence: thomas.lecuit@univ-amu.fr

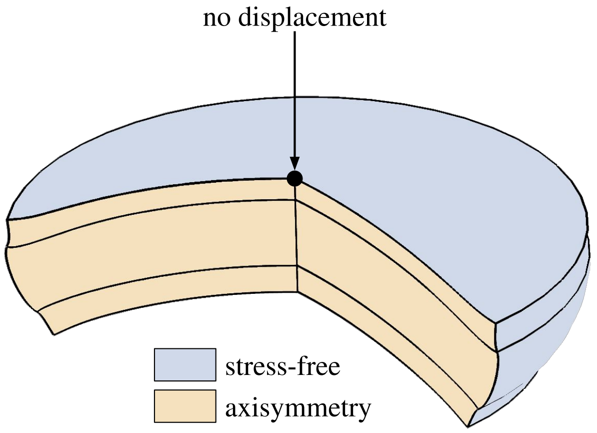

Figure M1: Boundary conditions for *Drosophila* wing disc sandwich model. The disc is assumed to be axisymmetric, with a no traction boundary condition $\mathbf{Tn} = 0$ imposed at the surface. The no-displacement boundary condition on one point of the symmetry axis (black dot) serves to eliminate translational degrees of freedom for the `Comsol` solver.

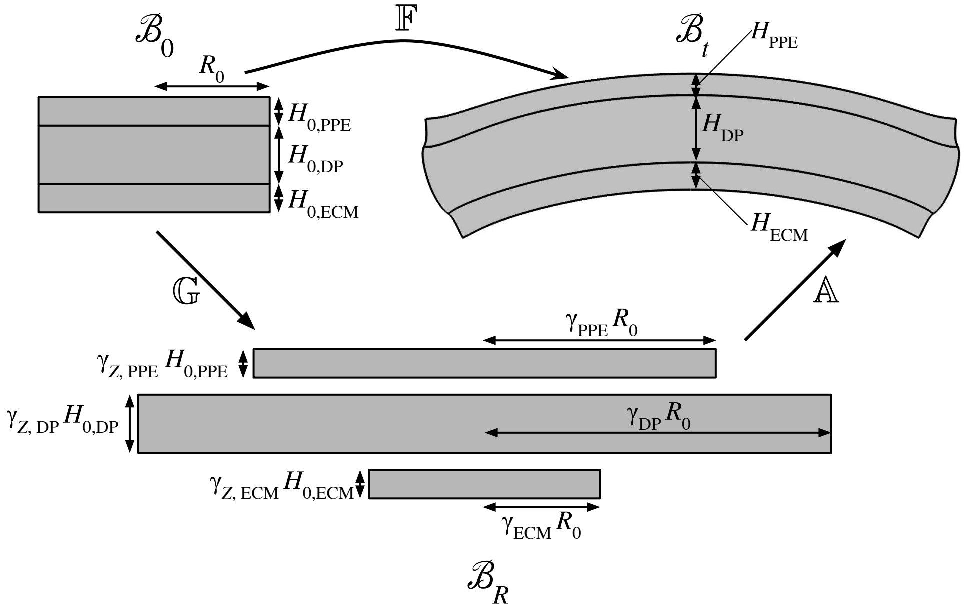

Figure M2: The wing disc is modelled as a multi-layer structure comprised of three layers or fewer. In the initial configuration $\mathcal{B}_0$, the tissue is ungrown and unstressed. The growth tensor $\mathbb{G}$ describes growth without stress, leading to an unstressed incompatible post-grown configuration $\mathcal{B}_R$, in which the individual layers have growth but do not fit into Euclidean space without breaking the connection between layers. Finally, the elastic deformation gradient $\mathbb{A}$ restores compatibility by introducing residual (internal) stress, bringing the body into the current (observed) configuration $\mathcal{B}_t$. The initial geometry in configuration $\mathcal{B}_0$ are three glued-together discs of radius R_0. The peripodial epithelium (PPE) is modelled as a disc of heigth H_{PPE}, the disc proper (DP) as a disc of heigth H_{DP}, and the extracellular matrix (ECM) as a disc with heigth H_{ECM}. In the post-grown configuration $\mathcal{B}_R$, the three discs have radius $\gamma_{Z,\,i} R_0$ and heigth $\gamma_{Z,\,i} H_i$ with $i \in \{\mathrm{PPE}, \mathrm{DP}, \mathrm{ECM}\}$, where the growth tensor in the cylindrical basis is $\mathbb{G} = \mathrm{diag}\,(\gamma_i, \gamma_i, \gamma_{Z,\,i})$.

growth is isotropic, and if $\gamma_i \neq \gamma_{Z,i}$, growth is anisotropic. Notice that we do not consider here the case of polar anisotropy, which means that we have implicitly assumed $\gamma_{R,i} = \gamma_{\theta,i} = \gamma_i$ in (M3). We denote $[\mathbb{G}]$ the components of $\mathbb{G}$ in the cylindrical basis:

$$[\mathbb{G}] = \mathrm{diag}\left(\gamma_i, \gamma_i, \gamma_{Z,i}\right), \qquad i \in \{\mathrm{PPE}, \mathrm{DP}, \mathrm{ECM}\}\ . \tag{M4}$$

The volume of the wing disc can be computed from the post-grown stress-free configuration $\mathcal{B}_R$, see Fig. M2. This is done by adding the volumes of the three flat discs in $\mathcal{B}_R$:

$$\mathrm{Volume} = \pi R_0^2 \left(H_{\mathrm{ECM}}\gamma_{\mathrm{ECM}}^2\gamma_{Z,\mathrm{ECM}} + H_{\mathrm{DP}}\gamma_{\mathrm{DP}}^2\gamma_{Z,\mathrm{DP}} + H_{\mathrm{PPE}}\gamma_{\mathrm{PPE}}^2\gamma_{Z,\mathrm{PPE}}\right)\ . \tag{M5}$$

The volume of the deformed configuration $\mathcal{B}_t$ will be the same in the limit $\kappa \to \infty$, which corresponds to an incompressible material ($|\mathbb{A}| = 1$).

M1.2 Three different scenarios

M1.2.1 Scenario: Bilayer PPE-DP

In this scenario, we test the hypothesis that the wing disc consists of two layers, a fast growing peripodial epithelium and a slower growing disc proper epithelium. The ECM is not modelled in this scenario, see Table M1 column "PPE vs DP". For the sake of simplicity, we consider planar growth in both PPE and DP, that is

$$[\mathbb{G}_{\mathrm{PPE}}] = \mathrm{diag}\left(\gamma_{\mathrm{PPE}}, \gamma_{\mathrm{PPE}}, 1\right), \qquad [\mathbb{G}_{\mathrm{DP}}] = \mathrm{diag}\left(\gamma_{\mathrm{DP}}, \gamma_{\mathrm{DP}}, 1\right)\ . \tag{M6}$$

The results of simulations with $\gamma_{\mathrm{PPE}} = 4.3$ and $\gamma_{\mathrm{DP}} = 3.31$, are shown in Fig. 3B. the relative volume increase of the PPE compared to the DP layer, normalized by initial volumes, is $|\mathbb{G}_{\mathrm{PPE}}|/|\mathbb{G}_{\mathrm{DP}}| = \gamma_{\mathrm{PPE}}^2/\gamma_{\mathrm{DP}}^2 = 1.69$, in other words the PPE layer grew 70% more in volume than the DP layer.

However, as discussed in the main text, experiments show that growth between PPE and DP is nearly compatible, demonstrate that epithelial doming and thickening are not due to a non-uniformity of growth within or between epithelial layers.

M1.2.2 Scenario: Bilayer DP-ECM, with differential growth anisotropy

In the main text, we describe how a two layer system made up of a DP layer and ECM layer capures the bending of the wing disc, as well as a number other of experimentally measured geometric quantities, see Fig. 5. In particular, the DP layer grows in plane whereas the ECM layer deviates from planar growth.

The growth tensors describing the two layers are

$$[\mathbb{G}_{\mathrm{DP}}] = \mathrm{diag}\left(\gamma_{\mathrm{DP}}, \gamma_{\mathrm{DP}}, 1\right), \qquad [\mathbb{G}_{\mathrm{ECM}}] = \mathrm{diag}\left(\gamma_{\mathrm{ECM}}, \gamma_{\mathrm{ECM}}, \gamma_{\mathrm{ECM}}^{\rho}\right)\ . \tag{M7}$$

The components of the growth tensor for the DP satisfy $\gamma_{\mathrm{DP}} = 1$ at $t = 65\mathrm{h}$ and $\gamma_{\mathrm{DP}} = \gamma_{\mathrm{DP}}^*$ at $t = 118\mathrm{h}$ and for the ECM they satisfy $\gamma_{\mathrm{ECM}} = 1$ at $t = 65\mathrm{h}$ and $\gamma_{\mathrm{ECM}} = \gamma_{\mathrm{ECM}}^*$ at $t = 118\mathrm{h}$.

For the disc proper, the parameter γ_{DP}^* can be determined by fitting the linear function

$$\gamma_{\mathrm{DP}}(t) = \frac{1}{53\mathrm{h}}\left[118\mathrm{h} + t\left(\gamma_{\mathrm{DP}}^* - 1\right) - 65\mathrm{h}\gamma_{\mathrm{DP}}^*\right]\ , \tag{M8}$$

which satisfies the above constraints. Since the volume over time in the DP, $V_{\mathrm{DP}}(t)$, is available from experiments, we now show how $\gamma_{\mathrm{DP}}(t)$ can be related to experimental volume measurements. The volume of the DP is given by $V_{\mathrm{DP}}(t) = V_{0,\mathrm{DP}}\,|\mathbb{G}_{\mathrm{DP}}|$, where $|\cdot| = \det(\cdot)$ denotes the determinant of a tensor. Here, $|\mathbb{G}_{\mathrm{DP}}| = \gamma_{\mathrm{DP}}^2(t)$. The last two equations can be combined to

$$V_{\mathrm{DP}}(t) = V_{0,\mathrm{DP}}\gamma_{\mathrm{DP}}^2(t)\ . \tag{M9}$$

The initial volume $V_{0,\mathrm{DP}}$ at $t = 65\mathrm{h}$ can be computed as the volume of a cylinder, $V_{0,\mathrm{DP}} = \pi R_0^2 H_{0,\mathrm{DP}}$ where the values for the initial radius, R_0, and initial heigth of the DP, $H_{0,\mathrm{DP}}$ are obtained from experimental measurements, as stated in Table M1 column "best fit DP vs ECM" (see the initial state, $\mathcal{B}_0$, in Fig. M2). With this information, we can solve (M9) for $\gamma_{\mathrm{DP}}(t) = \sqrt{V_{\mathrm{DP}}(t)/V_{0,\mathrm{DP}}}$. We make a least squares fit, using the experimentally measured values $V_{\mathrm{DP}}(t)$ and $V_{0,\mathrm{DP}}$ and using the form of (M8) for $\gamma_{\mathrm{DP}}(t)$. As a result of the least squares fit, we obtain $\gamma_{\mathrm{DP}}^* = 8.09$.

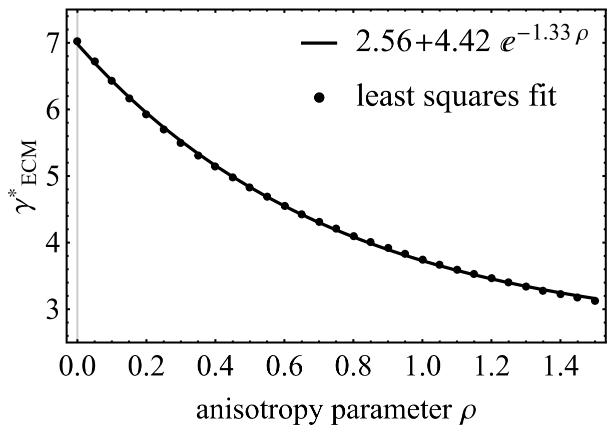

Figure M3: Obtaining a relationship between γ^*_{ECM} and ρ, using volumetric data of the ECM. In the in-plane scenario $\rho = 0$, all of the volume increase in the ECM is assumed to be distributed in the plane, and the in-plane growth component γ^*_{ECM} is highest in this case. As ρ increases, an increased amount of the measured volume is assumed to be distributed in Z-direction, so that the in-plane growth component γ^*_{ECM} decreases. The special case $\rho = 1$ represents isotropic growth, in which case the ECM growth tensor takes the form $\mathbb{G}_{\mathrm{ECM}} = \gamma_{\mathrm{ECM}}\mathbb{1}$, where $\mathbb{1}$ is the 3-dimensional identity.

To obtain γ^*_{ECM}, we proceed similarly. We use a linear functional form for $\gamma_{\mathrm{ECM}}(t)$, which is identical to (M8) (with the subscript DP replaced by ECM). The ECM volume, on the other hand, depends on ρ, since $|\mathbb{G}_{\mathrm{ECM}}| = \gamma^{2+\rho}_{\mathrm{ECM}}(t)$. Thus, the ECM volume is given by

$$V_{\mathrm{ECM}}(t) = V_{0,\mathrm{ECM}}\gamma^{2+\rho}_{\mathrm{ECM}}(t) \ , \tag{M10}$$

where $V_{0,\mathrm{ECM}} = \pi R_0^2 H_{0,\mathrm{ECM}}$ and values for R_0, $H_{0,\mathrm{ECM}}$ are stated in Table M1 column "best fit DP vs ECM". With this information, we can solve (M10) for $\gamma_{\mathrm{ECM}}(t) = (V_{\mathrm{ECM}}(t)/V_{0,\mathrm{ECM}})^{\frac{1}{2+\rho}}$. So for a given value of ρ, we make a least squares fit from experimental values $V_{\mathrm{ECM}}(t)$ and $V_{0,\mathrm{ECM}}$ and using the linear form for $\gamma_{\mathrm{ECM}}(t)$ given above. We repeat the least squares fitting for a series of values of ρ, obtaining data points $(\rho, \gamma^*_{\mathrm{ECM}})$ from the best fits, see Fig. M3. This data is well described by the smooth function $\gamma^*_{\mathrm{ECM}}(\rho) = 2.56 + 4.42e^{-1.33\rho}$.

With the parameters γ^*_{DP}, γ^*_{ECM} determined from volume data, the remaining parameters ρ and $\mu = \mu_{\mathrm{DP}}/\mu_{\mathrm{ECM}}$ are determined in Fig. 5B. There, we can see three distinct (partly overlapping) regions: In the blue region, labeled tol(H_{ECM}) $\leq$ 22.4%, the relative error between the mean experimentally measured value of ECM thickness and the simulation result for the corresponding pair of $\{\rho, \mu_{\mathrm{ECM}}/\mu_{\mathrm{DP}}\}$ values does not exceed 22.4%. Similarly, the rose region labeled tol(H_{DP}) $\leq$ 22.4% compares in the same way experimental vs. simulated disc proper thickness. Finally, the orange region labeled tol($\Delta H/H_{\mathrm{ECM}}$) $\leq$ 3.53% considers the relative thickness increase after vs before decellularization, that is the ratio $H^{\text{after decell}}_{\mathrm{ECM}}/H^{\text{before decell}}_{\mathrm{ECM}}$. The same ratio can be relatively easily considered in the model, where ratio of reference ECM thickness to observed ECM thickness is $\gamma^{\rho}_{\mathrm{ECM}}H_{0,\mathrm{ECM}}/H_{\mathrm{ECM}}$ (see Fig. 5a "reference state" and "observed state"). So in summary, in the orange region, the relative error between the mean experimentally measured value $H^{\text{after decell}}_{\mathrm{ECM}}/H^{\text{before decell}}_{\mathrm{ECM}}$ and the simulation result $\gamma^{\rho}_{\mathrm{ECM}}H_{0,\mathrm{ECM}}/H_{\mathrm{ECM}}$ for the corresponding pair of $\{\rho, \mu_{\mathrm{ECM}}/\mu_{\mathrm{DP}}\}$ values does not exceed 3.53%.

In Fig. 5b, we can see that these three regions overlap in the dark shaded region, where the disc proper thickness, ECM thickness and ECM relative thickness increase upon decellularization are all simultaneously within the tolerance values when experimental and simulation results are compared. This region allows us to determine the best fit $\rho = 0.45$ and $\mu_{\mathrm{ECM}}/\mu_{\mathrm{DP}} = 25$.

M1.2.3 Scenario: Bilayer DP-ECM, both layers growing in-plane

This scenario serves as a demonstration that differential growth anisotropy is indeed essential to capture the wing disc morphology. In Supplementary Fig. 8e, using the parameter values given in Table M1 column "in-plane DP vs ECM", we explore the scenario from Section M1.2.2 but with a crucial difference: There is no differential growth anisotropy ($\rho = 0$), meaning that both the DP and ECM grow in-plane. The result is shown in In Supplementary Fig. 8e, demonstrating clearly that without growth anisotropy, the correct wing disc morphology can not be achieved, we end up with a structure that is far too flat.

M2 Numerical implementation

Numerical simulations are obtained using a finite element code that solves the equation of finite elasticity on a multi-layer structure of glued-together axisymmetric cylinders. The 2D axisymmetric numerical problem is solved in Comsol Multiphysics® [3]. In Comsol, the morphoelatic decomposition $\mathbb{F} = \mathbb{A}\mathbb{G}$ cannot be directly entered into the software. We give here a succinct summary of the implementation procedure stated in works of Larry Taber [7, 12], showing how growth problems can be studied in Comsol.

M2.1 General case

Comsol computes derivatives with respect to displacement gradients to obtain a second Piola-Kirchhoff stress tensor

$$\overline{\mathbb{S}} = 2\frac{\partial W}{\partial \mathbb{C}_{\mathbb{F}}}\,, \tag{M11}$$

where we denote $\mathbb{C}_{\mathbb{F}} = \mathbb{F}^{\mathsf{T}}\mathbb{F}$ the right Cauchy-Green strain tensor of the total deformation gradient $\mathbb{F}$. In particular, the finite element formulation requires the stress $\overline{\mathbb{S}}$ per unit initial area ($\mathcal{B}_0$). The second Piola-Kirchhoff stress in terms of Cauchy stress is given by

$$\mathbb{S} = |\mathbb{F}|\,\mathbb{F}^{-1}\mathbb{T}\mathbb{F}^{-\mathsf{T}}\,. \tag{M12}$$

For an incompressible or nearly incompressible material, the Cauchy stress is given by (M2), which can be rewritten as

$$\mathbb{T} = \frac{2}{|\mathbb{A}|}\mathbb{A}\frac{\partial W}{\partial \mathbb{C}_{\mathbb{A}}}\mathbb{A}^{\mathsf{T}}\,, \tag{M13}$$

where we denote $\mathbb{C}_{\mathbb{A}} = \mathbb{A}^{\mathsf{T}}\mathbb{A}$ the right Cauchy-Green strain tensor of the elastic deformation gradient $\mathbb{A}$. Inserting (M13) into (M12), we get

$$\mathbb{S} = 2\frac{|\mathbb{F}|}{|\mathbb{A}|}\mathbb{G}^{-1}\frac{\partial W}{\partial \mathbb{C}_{\mathbb{A}}}\mathbb{G}^{-\mathsf{T}}\,. \tag{M14}$$

As shown in [7], the gradient of W satisfies the following transformation relationship:

$$\frac{\partial W}{\partial \mathbb{C}_{\mathbb{A}}} = \mathbb{G}\frac{\partial W}{\partial \mathbb{C}_{\mathbb{F}}}\mathbb{G}^{\mathsf{T}}\,. \tag{M15}$$

Inserting this into (M14), we find

$$\mathbb{S} = \frac{|\mathbb{F}|}{|\mathbb{A}|}\overline{\mathbb{S}}\,. \tag{M16}$$

So the appropriate expression of the second Piola-Kirchhoff stress can be obtained by multiplying the equation for $\overline{\mathbb{S}}$, which Comsol uses by default, by $|\mathbb{F}|\,/\,|\mathbb{A}|$. In addition, W must be defined in Comsol in terms of the components of $\mathbb{C}_{\mathbb{A}}$, that is

$$W\left(\mathbb{C}_{\mathbb{A}}\right) = W\left(\mathbb{G}^{-\mathsf{T}}\mathbb{C}_{\mathbb{F}}\mathbb{G}^{-1}\right)\,. \tag{M17}$$

.

M2.2 Specific case

Note that Comsol will solve for $\mathbb{C}_{\mathbb{F}}$. Therefore, it is necessary to provide Comsol with the components of $\mathbb{C}_{\mathbb{A}}$ in terms of the components of $\mathbb{C}_{\mathbb{F}}$, in order for a successful implementation of the modified strain energy W and the modification (M16). In our problem, we assume that the growth tensor is written in a cylindrical basis $\{\mathbf{E}_R, \mathbf{E}_\theta, \mathbf{E}_Z\}$ where it has a diagonal form

$$[\mathbb{G}] = \operatorname{diag}\left(\gamma_R, \gamma_\theta, \gamma_Z\right)\,. \tag{M18}$$

		PPE vs DP (Fig. 2)	best fit DP vs ECM (Fig. 5)	in-plane DP vs ECM (SI Fig. S8e)	reference
geometric	R_0 [μm]	20.05	20.05	20.05	measured
	$H_{0,\mathrm{PPE}}$ [μm]	5.25	-	-	measured
	$H_{0,\mathrm{DP}}$ [μm]	22.36	22.36	22.36	measured
	$H_{0,\mathrm{ECM}}$ [μm]	-	3.09	3.09	measured
stiffnes	μ_{PPE} [kPa]	1	-	-	refs [11, 4, 10]
	μ_{DP} [kPa]	1	1	1	
	μ_{ECM} [kPa]	-	25	25	Fig. 5b
growt at 118h	γ_{PPE} [1]	3	-	-	-
	γ_{DP} [1]	2.5	8.09	8.09	measured volumes
	γ_{ECM} [1]	-	4.99	6.99	measured volumes
	ρ	-	0.45	0	Fig. 5b

Table M1: Simulation parameter values for the different scenarios described in Sec. M1.2.

Furthermore, the components $[\mathbb{C}_\mathbb{F}]_{R\theta}$, $[\mathbb{C}_\mathbb{F}]_{\theta Z}$ are fixed by the constraint of axisymmetry. Further recalling that $\mathbb{C}_\mathbb{A}$ is symmetric, the four unique components $[\mathbb{C}_\mathbb{A}]$ are for this particular geometry:

$$[\mathbb{C}_\mathbb{A}]_{RR} = \frac{[\mathbb{C}_\mathbb{F}]_{RR}}{\gamma_R^2}\,, \tag{M19}$$

$$[\mathbb{C}_\mathbb{A}]_{RZ} = \frac{[\mathbb{C}_\mathbb{F}]_{RZ}}{\gamma_R \gamma_Z}\,, \tag{M20}$$

$$[\mathbb{C}_\mathbb{A}]_{\theta\theta} = \frac{[\mathbb{C}_\mathbb{F}]_{\theta\theta}}{\gamma_\theta^2}\,, \tag{M21}$$

$$[\mathbb{C}_\mathbb{A}]_{ZZ} = \frac{[\mathbb{C}_\mathbb{F}]_{ZZ}}{\gamma_Z^2}\,. \tag{M22}$$

These can be inserted into the re-defined strain energy density for Comsol, (M17), and used to compute the determinant $|\mathbb{A}|$ for the re-defined second Piola Kirchhoff stress (M16).

M3 Validation of the numerical implementation against exact solution

In this section, we test the computational framework presented in Section M2 by comparing it with an analytically known solution for an incompressible neo-Hookean material that grows. The setup we are considering is shown in Fig. M4A. We consider a cylinder with an initial radius R_0, with no displacement allowed in Z-direction, that is $[\mathbb{F}]_{ZZ} = 1$. In the analytical axisymmstric calculation, we impose $[\mathbb{F}]_{ZZ} = 1$ everywhere in the bulk, whereas in the numerical implementation, as shown in Fig. M4A, we impose this constraint on the top and bottom highlighted planes. Further, we impose a polar growth anisotropy. Our goal is to compare the numerical and analytical expressions for the Cauchy stress tensor and to determine the relative error between them, so that we establish a benchmark for what kind of error to expect in the simulations presented in Section M1.2 where no analytical solution exists.

M3.1 Derivation of exact solution

We consider the case of a single incompressible growing neo-Hookean disc. We assume that there is no deformation at the point of symmetry, and that there are no external forces, so that any deformation is caused purely by growth and the elastic response. This calculation follows a similar path to [5, 2, 6]. For an incompressible disc in the cylindrical basis $\{\mathbf{E}_R, \mathbf{E}_\theta, \mathbf{E}_Z\}$, the morphoelastic decomposition $\mathbb{F} = \mathbb{A}\mathbb{G}$

reads

$$[\mathbb{F}] = \begin{pmatrix} r'(R) & 0 & 0 \\ 0 & \frac{r}{R} & 0 \\ 0 & 0 & 1 \end{pmatrix}, \qquad [\mathbb{A}] = \begin{pmatrix} \alpha_R & 0 & 0 \\ 0 & \alpha_\theta & 0 \\ 0 & 0 & 1 \end{pmatrix} = \begin{pmatrix} \alpha^{-1} & 0 & 0 \\ 0 & \alpha & 0 \\ 0 & 0 & 1 \end{pmatrix}, \qquad [\mathbb{G}] = \begin{pmatrix} \gamma_R & 0 & 0 \\ 0 & \gamma_\theta & 0 \\ 0 & 0 & 1 \end{pmatrix}. \tag{M23}$$

Eliminating α, we obtain the kinematic relationship

$$r(R)\, r'(R) = \gamma_R \gamma_\theta R, \qquad r(0) = 0\,. \tag{M24}$$

Let $W(\alpha_R, \alpha_\theta)$ be the strain-energy density, which relates to the Cauchy stress tensor by

$$\mathbb{T} = \frac{\partial W}{\partial \mathbb{A}} \mathbb{A}^{\mathsf{T}} - p\mathbb{1}, \tag{M25}$$

where p is the Lagrange multiplier enforcing incompressibility. In components this reads

$$T_R = \alpha_R \frac{\partial W}{\partial \alpha_R} - p, \qquad T_\theta = \alpha_\theta \frac{\partial W}{\partial \alpha_\theta} - p\,, \tag{M26}$$

where we used the shorthand notation $[\mathbb{T}]_{RR} = T_R$, $[\mathbb{T}]_{\theta\theta} = T_\theta$. With no external loads, mechanical equilibrium requires div $\mathbb{T} = 0$, which takes the form $\partial T_R / \partial r = (T_\theta - T_R)/r$. Expressing with respect to the reference radius R, this becomes

$$\frac{\partial T_R}{\partial R} = \frac{r'}{r}(T_\theta - T_R), \qquad T_R(B) = 0\,. \tag{M27}$$

Defining $\widehat{W}(\alpha) := W\left(\alpha^{-1}, \alpha\right)$, we have

$$T_\theta - T_R = \alpha \widehat{W}'(\alpha)\,. \tag{M28}$$

Now taking into account (M28), for the radial stress we must solve

$$\frac{\partial T_R}{\partial R} = \frac{\gamma_R}{r} \widehat{W}'\left(\frac{r}{R\gamma_\theta}\right), \qquad T_R(B) = 0\,. \tag{M29}$$

For a neo-Hookean strain-energy density

$$W(\alpha_R, \alpha_\theta) = \frac{\mu}{2}\left(\alpha_R^2 + \alpha_\theta^2 - 2\right), \tag{M30}$$

and taking into account the relationships from the morphoelastic decomposition (M2) and (M24), we can express (M29) as

$$\frac{\partial T_R}{\partial_R} = \frac{\mu \gamma_R}{\gamma_\theta R}\left(1 - \frac{R^4 \gamma_\theta^4}{r^4}\right), \qquad T_R(B) = 0\,. \tag{M31}$$

Once the radial stress is known, the circumferential stress can be obtained from (M28)

$$T_\theta = T_R + \alpha \widehat{W}'(\alpha) = T_R + \frac{\mu r^2}{\gamma_\theta^2 R^2}\left(1 - \frac{\gamma_\theta^4 R^4}{r^4}\right). \tag{M32}$$

To obtain the full solution, one must provide $\gamma_R(R)$ and $\gamma_\theta(R)$ and can then solve the two non-linear coupled ODEs (M28) and (M31). For γ_R, γ_θ spatially constant, we obtain the exact analytical solution

$$r(R) = \sqrt{\gamma_R \gamma_\theta} R \tag{M33}$$

$$T_R(R) = \mu\left(\frac{\gamma_R}{\gamma_\theta} - \frac{\gamma_\theta}{\gamma_R}\right) \log\left(\frac{R}{B}\right) \tag{M34}$$

$$T_\theta(R) = \mu\left(\frac{\gamma_R}{\gamma_\theta} - \frac{\gamma_\theta}{\gamma_R}\right)\left[\log\left(\frac{R}{B}\right) + 1\right]. \tag{M35}$$

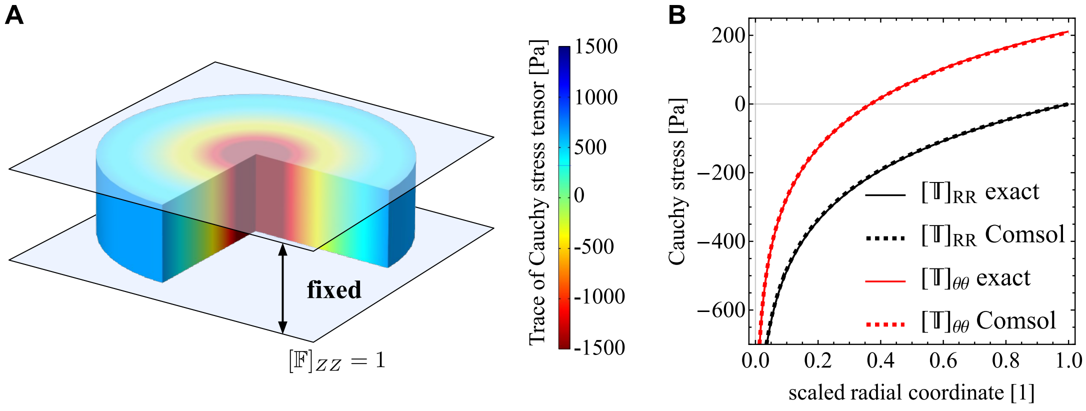

Figure M4: Comparison between analytical solution and numerical implementation in Comsol.

M3.2 Comparison of exact and analytical solutions

In Fig. M4, we compare the exact analytical solution (M34), (M35) with the numerical implementation in Comsol Multiphysics® as described in Section M2. We obtain excellent agreement, with the largest relative error between numerics and analytics at the disc center being 3.36% and the larges error at the disc edge being 1.38%. Given that at the disc center, the stress tensor has a singularity ($T \sim \log R$) as see in (M34) and (M35), we expect the lower error at the edge to be more representative. The Finite Element mesh in this case was composed of 26006 triangular elements (all simulations presented in Section M1.2 had around 25k triangular elements, always using the Comsol predefined setting "Extremely fine" for the mesh, without any manually defined mesh refinement). Overall, the comparison between exact and analytical solutions shows that the numerical implementation is reliable, and justifies its use for more complex geometries like the sandwich structures presented in the paper.

Supplementary References

[1] D. Ambrosi and F. Guana. Stress-Modulated Growth. *Mathematics and Mechanics of Solids*, 12(3):319–342, 11 2005.

[2] Davide Ambrosi, Viola Pettinati, and Pasquale Ciarletta. Active stress as a local regulator of global size in morphogenesis. *International Journal of Non-Linear Mechanics*, 75:5–14, 2015.

[3] Stockholm COMSOL AB. Comsol multiphysics® v. 4.3, 2012.

[4] Maria Duda, Natalie J Kirkland, Nargess Khalilgharibi, Melda Tozluoglu, Alice C Yuen, Nicolas Carpi, Anna Bove, Matthieu Piel, Guillaume Charras, Buzz Baum, et al. Polarization of myosin ii refines tissue material properties to buffer mechanical stress. *Developmental cell*, 48(2):245–260, 2019.

[5] Alexander Erlich, Derek E Moulton, and Alain Goriely. Are homeostatic states stable? dynamical stability in morphoelasticity. *Bulletin of mathematical biology*, 81(8):3219–3244, 2019.

[6] Alain Goriely. *The mathematics and mechanics of biological growth*, volume 45. Springer, 2017.

[7] Hadi S Hosseini, David C Beebe, and Larry A Taber. Mechanical effects of the surface ectoderm on optic vesicle morphogenesis in the chick embryo. *Journal of biomechanics*, 47(16):3837–3846, 2014.

[8] Thomas J Pence and Kun Gou. On compressible versions of the incompressible neo-hookean material. *Mathematics and Mechanics of Solids*, 20(2):157–182, 2015.

[9] Edward K Rodriguez, Anne Hoger, and Andrew D McCulloch. Stress-dependent finite growth in soft elastic tissues. *Journal of biomechanics*, 27(4):455–467, 1994.

[10] Thomas Schluck. *Mechanical perturbation and stimulation of drosophila wing imaginal discs*. PhD thesis, University of Zurich, 2013.

[11] Thomas Schluck and Christof M Aegerter. Photo-elastic properties of the wing imaginal disc of drosophila. *The European Physical Journal E*, 33(2):111–115, 2010.

[12] Larry A Taber. Theoretical study of beloussovś hyper-restoration hypothesis for mechanical regulation of morphogenesis. *Biomechanics and modeling in mechanobiology*, 7(6):427–441, 2008.

[13] Gang Xu, Philip V Bayly, and Larry A Taber. Residual stress in the adult mouse brain. *Biomechanics and modeling in mechanobiology*, 8(4):253–262, 2009.
