# Supplementary material for: Growth anisotropy of the extracellular matrix shapes a developing organ
Source: Nat Commun. 2023 Mar 3;14:1220. doi: 10.1038/s41467-023-36739-y (PMC9984492; doi:10.1038/s41467-023-36739-y)
Supplement: Supplementary file 3 — Reporting Summary [file 41467_2023_36739_MOESM3_ESM.pdf]

nature portfolio

Corresponding author(s): Thomas Lecuit

Last updated by author(s): Feb 9, 2023

Reporting Summary

Nature Portfolio wishes to improve the reproducibility of the work that we publish. This form provides structure for consistency and transparency in reporting. For further information on Nature Portfolio policies, see our Editorial Policies and the Editorial Policy Checklist.

Statistics

For all statistical analyses, confirm that the following items are present in the figure legend, table legend, main text, or Methods section.

n/a	Confirmed	
☐	☒	The exact sample size (*n*) for each experimental group/condition, given as a discrete number and unit of measurement
☐	☒	A statement on whether measurements were taken from distinct samples or whether the same sample was measured repeatedly
☐	☒	The statistical test(s) used AND whether they are one- or two-sided<br>*Only common tests should be described solely by name; describe more complex techniques in the Methods section.*
☒	☐	A description of all covariates tested
☒	☐	A description of any assumptions or corrections, such as tests of normality and adjustment for multiple comparisons
☐	☒	A full description of the statistical parameters including central tendency (e.g. means) or other basic estimates (e.g. regression coefficient) AND variation (e.g. standard deviation) or associated estimates of uncertainty (e.g. confidence intervals)
☐	☒	For null hypothesis testing, the test statistic (e.g. *F*, *t*, *r*) with confidence intervals, effect sizes, degrees of freedom and *P* value noted<br>*Give P values as exact values whenever suitable.*
☒	☐	For Bayesian analysis, information on the choice of priors and Markov chain Monte Carlo settings
☒	☐	For hierarchical and complex designs, identification of the appropriate level for tests and full reporting of outcomes
☒	☐	Estimates of effect sizes (e.g. Cohen's *d*, Pearson's *r*), indicating how they were calculated

Our web collection on statistics for biologists contains articles on many of the points above.

Software and code

Policy information about availability of computer code

Data collection	Leica Application Suite X 3.55.19976, Metamorph 7.8.4.0, Nikon NIS-Elements AR 5.11.01
Data analysis	Fiji / imageJ 1.53t (National Institute of Health), Anaconda Navigator 2.1.1 / Spyder 5.1.5 / Python 3.9.7, Ilastik 1.3.3, Seaborn 0.11.2<br>The code used for data analysis, simulations, and model fitting is available from the corresponding author [T.L.] on request.

For manuscripts utilizing custom algorithms or software that are central to the research but not yet described in published literature, software must be made available to editors and reviewers. We strongly encourage code deposition in a community repository (e.g. GitHub). See the Nature Portfolio guidelines for submitting code & software for further information.

Data

Policy information about availability of data

All manuscripts must include a data availability statement. This statement should provide the following information, where applicable:

- Accession codes, unique identifiers, or web links for publicly available datasets
- A description of any restrictions on data availability
- For clinical datasets or third party data, please ensure that the statement adheres to our policy

The data supporting the findings of this study and materials are available on request from the corresponding author [T.L.].

Human research participants

Policy information about studies involving human research participants and Sex and Gender in Research.

Reporting on sex and gender	N/A
Population characteristics	N/A
Recruitment	N/A
Ethics oversight	N/A

Note that full information on the approval of the study protocol must also be provided in the manuscript.

Field-specific reporting

Please select the one below that is the best fit for your research. If you are not sure, read the appropriate sections before making your selection.

☒ Life sciences ☐ Behavioural & social sciences ☐ Ecological, evolutionary & environmental sciences

For a reference copy of the document with all sections, see nature.com/documents/nr-reporting-summary-flat.pdf

Life sciences study design

All studies must disclose on these points even when the disclosure is negative.

Sample size	Given the experimental constraints we aimed to obtain a sample size large enough (n ≥ 5) to allow testing statistical significance (see methods section 10).
Data exclusions	Samples (wing discs) that were obviously damaged during the dissection and mounting procedure were excluded due to their aberrant morphology.
Replication	Experiments were performed at least twice independently during the establishment of the experimental procedures and consistently resulted in the same qualitative outcome. The final set of experiments were carefully analyzed and statistically evaluated. For each experiment the number of independent biological replicates (e.g. wing discs) is indicated.<br>Detailed information is given in the methods section that allows replication of all the manipulations shown in this study.
Randomization	No randomization was done, however all larvae of an experiment were kept in the same incubator, as well as dissected and processed together using identical solutions in order to minimize variation between the different experimental groups. Also, quantitative data sets were imaged together in one session using identical imaging settings.
Blinding	Blinding was not possible due to the obvious phenotypes observed.

Reporting for specific materials, systems and methods

We require information from authors about some types of materials, experimental systems and methods used in many studies. Here, indicate whether each material, system or method listed is relevant to your study. If you are not sure if a list item applies to your research, read the appropriate section before selecting a response.

Materials & experimental systems

n/a	Involved in the study	
☐	☒	Antibodies
☒	☐	Eukaryotic cell lines
☒	☐	Palaeontology and archaeology
☐	☒	Animals and other organisms
☒	☐	Clinical data
☒	☐	Dual use research of concern

Methods

n/a	Involved in the study	
☒	☐	ChIP-seq
☒	☐	Flow cytometry
☒	☐	MRI-based neuroimaging

Antibodies

Antibodies used	Primary antibodies used were mouse-anti-Wingless (4D4-s; 1:120; DSHB, University of Iowa); mouse-anti-Patched (Apa1-s; 1:40;

Antibodies used	DSHB, University of Iowa); rat-anti-DE-cadherin (DCAD2 concentrate; 1:200; DSHB, University of Iowa); rabbit-anti-GFP (1:1000, Abcam ab6556, Lot:GR3404234-1); rabbit-anti-Phospho-Histone H3 (PHH3, 1:1000, Cell Signaling #9701); rabbit-anti-Mmp2 (1:500, from K. Broadie Ref.67), rabbit-anti-Vkg (1/500, from S. Noselli, Ref.68). Secondary antibodies used were Alexa 488 (donkey anti-mouse A21202, donkey anti-rabbit A21206), Alexa 568 (donkey anti-mouse A10037, donkey anti-rabbit A10042, goat anti-rat A11077) and Alexa 647 (donkey anti-rabbit A31573) from Invitrogen and Alexa 647 (donkey anti-mouse 715 605 151) from Jackson ImmunoResearch.
Validation	All the utilized antibodies have been validated and used extensively in multiple studies. For DSHB antibodies see https://dshb.biology.uiowa.edu/ (4D4 – 68 citations, Apa1 – 20 citations, DCAD2 – 47 citations). For the Abcam rabbit anti-GFP see https://www.abcam.com (ab 6556 - 1073 citations). For Cell Signaling rabbit-anti-Phospho-Histone H3 see https://www.cellsignal.com (#9701 – 2230 citations). The rabbit-anti-Vkg antibody has been shown to specifically mark Collagen IV in Reference 68. Rabbit-anti-Mmp2 was previously used in Drosophila larval discs (eye and wing disc). We further ensured its specificity by showing a clear reduction of MMP2 signal upon MMP2 knock-down (see Supplementary Fig.8).

Animals and other research organisms

Policy information about studies involving animals; ARRIVE guidelines recommended for reporting animal research, and Sex and Gender in Research

Laboratory animals	Drosophila melanogaster. Age of animals was determined in hours after egg laying (hAEL) as described in the methods. Only male larvae were included in the study to reduce variation (due to different growth dynamics in male and female larvae).<br><br>The following fly lines were used: y1,w1118, hs-Flp; act>Stop>Gal4, UAS-EGFP (AyGAL4, originating from Bloomington stock 64231); UAS-Histone::mRFP 70, VkgG454::GFP 47 (both from F. Schnorrer), UAS-MMP2, vgQE-dsRed 71, sqh-Sqh::mCherry 72 (insertion site 53B2), endo-Ecad::GFP 73. The following lines were obtained from the Bloomington stock centre: AGiR-Gal4 (#6773), UAS-PI3KDN (#25918), UAS-CD8::mRFP (#27398), UAS-CD8::RFP (#27392), UAS-Mmp2 TRiP (#61309), Mi{MIC} insertion in Mmp2 (#60512). hh::Gal4 is described on FlyBase (www.flybase.org).<br><br>Precise genotyes are indicated in methods section 2.
Wild animals	No wild animals used
Reporting on sex	As indicated above, only male animals were included in this study
Field-collected samples	No field-collected samples in this study
Ethics oversight	Studies in invertebrates do not require ethical approval in France.

Note that full information on the approval of the study protocol must also be provided in the manuscript.
